# Isovaleric Acidemia in Jordan

**DOI:** 10.7759/cureus.52039

**Published:** 2024-01-10

**Authors:** Noor Megdadi, Mo'men Alakil, Lina Ghanmiyin, Omar Maaita, Amjad Abulannaz

**Affiliations:** 1 Department of Pediatrics, Royal Medical Services, Amman, JOR

**Keywords:** jordan, inborn error of metabolism, acidurias, organic acidemia, isovaleric acidemia

## Abstract

Background: Isovaleric acidemia (IVA) was the first condition to be recognized as an organic acid disorder. It is marked by metabolic ketoacidosis with an unexplained anion gap. This study examines IVA in Jordan, laying the groundwork for future studies. Furthermore, it seeks to enhance the understanding of clinical characteristics and outcomes in affected individuals.

Method: This case series study includes all isovaleric acidemia diagnoses at the metabolic unit of the Queen Rania Al Abdullah Hospital for Children (QRHC) in Amman, Jordan, from 2010 to 2023. The study encompassed sociodemographic features, clinical and laboratory results, familial history, and parental consanguinity.

Results: Our cohort was composed of 21 individuals (10 males and 11 females), who presented IVA at an average age of 3.1 years. Positive family history and parental consanguinity were observed in 23.8% and 75% of the cases, respectively. Vomiting was the most prevalent symptom (57.1%), and encephalopathy occurred in 33.3%. Laboratory results showed acidosis (81%), hyperammonemia (71.4%), and hypoglycemia (14.3%).

Conclusions: The early initiation of treatment for organic acid disorders carries a more favorable prognosis. Therefore, we strongly recommend for implementing newborn screening to overcome diagnostic challenges and delays. For effective intervention, healthcare professionals should have a comprehensive understanding of the clinical manifestations of IVA and be proficient in interpreting biochemical test results.

## Introduction

Isovaleric acidemia (IVA) was the first condition to be recognized as an organic acid disorder. It is diagnosed in infants with acute episodic encephalopathy, characterized by a "sweaty feet" odor caused by an accumulation of unconjugated isovaleric acid due to a defect in isovaleryl-coenzyme A (CoA) dehydrogenase (IVD) [1,2]. Neurocognitive development is more strongly influenced by age at diagnosis than by the number or severity of metabolic decompensations. IVA's effects on neurocognitive development are generally mild, with 62% attaining normal or near-normal functioning [3]. The frequency of metabolic decompensation decreases with age, possibly due to acquired immunity [1]. Comorbidities include failure to thrive, myeloproliferative syndrome, pancytopenia, seizures, liver dysfunction, cardiopulmonary disorders, and pancreatitis [4-7].

IVA is characterized by metabolic ketoacidosis with an unexplained anion gap, detected through gas chromatography/mass spectrometry (GC/MS), revealing elevated isovalerylglycine and 3-hydroxyisovaleric acid. Isovaleryl-CoA dehydrogenase deficiency leads to isovaleryl-CoA accumulation during metabolic decompensation; this excess inhibits N-acetylglutamate synthetase, causing urea cycle impairment and hyperammonemia. Isovalerylcarnitine elevation may also indicate IVA. Confirmation involves biochemical analysis and assessing IVD enzyme activity [8,9]. Newborn screening allows early detection, enabling proactive intervention and reducing symptom severity, morbidity, and mortality. Early diagnosis is crucial, even for forms of IVA manifesting later in childhood [10].

The core treatment for organic acid disorders involves protein restriction, glycine/carnitine supplementation, and a leucine-restricted metabolic formula. Preventive measures for childhood illnesses, coupled with prompt emergency protocols during metabolic decompensations, are crucial in preventing adverse outcomes, reducing morbidity, and lowering mortality in organic acid disorders [11,12].

This study examines IVA in Jordan, establishing a database for subsequent studies. Additionally, it enhances the understanding of clinical characteristics and outcomes in affected individuals.

## Materials and methods

In this case series, we examined all patients treated for isovaleric acidemia at the Queen Rania Al Abdullah Hospital for Children (QRHC) from 2010 to 2023. QRHC is an integrated hospital within King Hussein Medical Center (KHMC) in Amman, the capital of Jordan. Its metabolic unit serves as a referral center for metabolic disorders from all districts of the country.

After obtaining approval from the Royal Medical Services Human Research Ethics Committee (approval number: 12/2023), we extracted patients' demographic details and clinical and laboratory data from metabolic unit records and the QRHC electronic archiving system. Information regarding patients' family history and parental consanguinity was obtained. The presented clinical features and laboratory results were then analyzed.

The study included patients with a diagnosis of isovaleric acidemia that was confirmed through an advanced diagnostic biochemical metabolic workup. This included the acylcarnitine profile in dry blood spots, analyzed by tandem mass spectrometry, and quantitative urine organic acid analysis conducted through gas chromatography/mass spectrometry. Suspected undiagnosed cases and unexplained metabolic deaths were excluded. Descriptive analysis, employing mean and standard deviation for continuous measures and frequency and percentages for categorical variables, was utilized to characterize the data comprehensively.

## Results

Our series included 21 patients (Table 1), composed of 10 males and 11 females. The mean age at presentation was 3.1 years (±2.9), and the mean age at the time of the study was seven years (±5.2). Positive family history was identified in 23.8% of the patients (five individuals), and parental consanguinity was observed in 16 (75.2%).

**Table 1:** Sociodemographic characteristics of IVA patients. IVA: isovaleric acidemia

	Number	Percentage
Sex		
Male	10	47.6
Female	11	52.4
Positive family history	5	23.8
Parental consanguinity	16	75.2

Table 2 shows the clinical presentation of IVA. Vomiting and the refusal of food were the most common symptoms at presentation, with encephalopathy reported in 12 patients (33.3%). Failure to thrive primarily involves insufficient physical growth, while developmental delay encompasses a broader range of delays in achieving developmental milestones across different domains, as reported in four (19%) and five (23.8%) patients, respectively. A child may experience developmental delays without necessarily failing to thrive and vice versa. Laboratory findings were demonstrated with the following frequencies: acidosis (17, 81%), hyperammonemia (15, 71.4%), and hypoglycemia (three, 14.3%).

**Table 2:** Presentation of IVA (N=21). IVA: isovaleric acidemia

	Number	Percentage
Metabolic acidosis	17	81
Hyperammonemia	15	71.4
Hypoglycemia	3	14.3
Encephalopathy	7	33.3
Vomiting and refusing food	12	57.1
Hypotonia	3	14.3
Failure to thrive	4	19
Developmental delay	5	23.8

Table 3 shows the case series for all 21 patients. One patient presented at the age of 2.5 years, was diagnosed with IVA, and died during admission. The analysis of acylcarnitines is crucial for assessing metabolic acidosis. These compounds play a key role in transporting fatty acids and eliminating organic acids. Carnitine supplementation is beneficial for eliminating metabolites in certain metabolic disorders. The nomenclature of acylcarnitines is determined by the number of carbons, double bonds, and the presence of hydroxyl groups. A high level ofIsovaleryl-/2-Methylbutyrulcarnitine (C5) was noted in all patients.

**Table 3:** Case series of 21 patients. *The patient passed at the age of 2.5. M, male; F, female; +, present; -, negative

Table 3: Case series of 21 patients.														
Case number	Age at presentation	Age at study	Sex	Family history	Parental consanguinity	Acidosis	Hyperammonemia	Hypoglycemia	Encephalopathy	vomiting	Hypotonia	Failure to thrive	Developmental delay
1	2	6.9	M	-	+	Yes	Yes	No	No	No	Yes	Yes	Yes
2	5	14	F	+	+	Yes	Yes	No	No	No	No	No	No
3	2	7.6	M	-	+	Yes	No	No	No	Yes	No	No	No
4	9	17	F	-	-	Yes	Yes	No	No	No	No	No	No
5	0.17	5.8	F	-	-	Yes	No	No	No	Yes	No	No	No
6	2.5	6	M	-	+	No	Yes	No	No	No	No	No	No
7	10	17.4	F	+	+	No	Yes	Yes	No	Yes	No	No	No
8	6	14	F	+	+	Yes	Yes	No	No	Yes	No	No	No
9	2.5	7.8	F	-	-	Yes	Yes	No	No	Yes	No	No	No
10	0.1	1.3	M	-	+	No	Yes	No	No	Yes	No	No	No
11	0.17	2	M	-	-	No	No	No	Yes	Yes	No	No	No
12	7	8.3	M	+	+	Yes	Yes	No	Yes	Yes	No	No	Yes
13	2	2.7	F	-	-	Yes	Yes	No	Yes	Yes	No	Yes	No
14	2	2.8	M	-	+	Yes	Yes	No	No	Yes	No	No	No
15	5	11.6	F	-	+	Yes	Yes	No	No	Yes	No	No	No
16	1	1.8	M	-	+	Yes	Yes	No	No	No	No	No	No
17	0.7	10	M	+	+	Yes	No	No	Yes	No	No	No	No
18	2.5	2.9	M	-	+	Yes	No	Yes	Yes	No	No	No	No
19	0.75	1.8	F	-	+	Yes	Yes	No	Yes	No	Yes	Yes	Yes
20	1.17	2.8	F	-	+	Yes	No	No	No	No	No	No	Yes
21*	2.5	2.5	F	-	-	Yes	Yes	Yes	Yes	Yes	Yes	Yes	Yes

## Discussion

The Queen Rania Al Abdullah Hospital for Children (QRHC) is an integrated facility within King Hussein Medical City in Amman. It is a specialized referral center for the entire country, including for metabolic diseases. Given the rarity of metabolic diseases and the need for specialized treatment, most cases are referred to our units, making studies from the metabolic unit at QRHC representative of the entire country of Jordan.

The exact incidence of metabolic diseases remains unexplored in Jordan. However, due to the high prevalence of consanguineous marriages, such conditions are expected to be high. Therefore, further studies are imperative to determine the prevalence of metabolic diseases, including IVA, in Jordan. Establishing a foundation for future investigations will make it possible to formulate a screening program for metabolic diseases accordingly.

A study from our center published in 2012 [13] reviewed the cases of 51 patients with organic acidemia treated at a metabolic clinic at KHMC over five years (2005-2010). Propionic acidemia was the most common type (14, 27.5%), followed by methylmalonic acidemia (six, 11.8%). In 29 patients (56.9%), the exact type remained undetermined. Nevertheless, they presented with acidotic breathing, with or without encephalopathy, and their blood gases revealed wide anion gap metabolic acidosis and elevated ammonia and lactate levels. Records in our clinic show 73 patients with organic acidemia, with IVA being the most common type, with 21 patients representing 28.8% of organic acidemia. The variation between recent and older reviews may be attributed to 56.9% of the organic acidemia subtype being undiagnosed in the previous study.

IVA manifests in two symptomatic forms: the common acute neonatal type, featuring metabolic ketoacidosis, and the chronic intermittent form, emerging later with developmental delay. Early neonatal onset carries a high mortality rate, but timely diagnosis markedly improves outcomes. IVA, like other organic acidemias, can present in newborns with symptoms such as irritability, lethargy, vomiting, seizures, altered consciousness, and even mortality. It may also mimic sepsis-like symptoms [14-16]. The older age at presentation in our unit may be attributed to delayed diagnosis among patients who received initial treatment at district hospitals in Jordan. Patients may not be accurately diagnosed due to the perplexing clinical presentation and the absence of organic acidemia, including the IVA, screening program in Jordan. In our study, the mean age at diagnosis or presentation was 3.1 years, primarily reflecting the chronic intermittent form. This suggests that the actual prevalence could be higher, given that many neonates passed without reaching diagnosis.

In IVA, 62% of the patients achieve normal or near-normal neurocognitive functioning with an early neonatal diagnosis, significantly improving outcomes [3]. A screening program that enables early diagnosis would allow treatment to begin sooner, minimizing complications.

There are certain limitations to our study. The lack of extensive clinical records hinders a comprehensive analysis of our findings. Patients might not have been referred to our facility, possibly due to early mortality or seeking treatment locally. Consequently, we expect that the true prevalence of IVA is higher than our study suggests. We recommend including organic acidemia in national screening programs to improve affected individuals' well-being and care outcomes.

## Conclusions

IVA is the predominant form of organic acidemia treated in the metabolic unit at the Queen Rania Al Abdullah Hospital for Children. Given the challenges and delays associated with diagnosing such conditions, a strong recommendation is made to introduce newborn screening as a key measure for early intervention and guidance. Moreover, healthcare professionals should have a comprehensive understanding of the clinical manifestations of these disorders and proficiency in interpreting biochemical test results.
